# Clinical outcomes of patients with pancreatic tumors discussed in Tumor Board

**DOI:** 10.1590/0100-6991e-20223150en

**Published:** 2022-04-13

**Authors:** JULIANA RIBEIRO SILVA, RACHEL SIMÕES PIMENTA RIECHELMANN, BÁRBARA ALANA VIZZACCHI, PATRICIA MOLINA, VICTOR HUGO FONSECA DE JESUS, FELIPE JOSE FERNANDEZ COIMBRA, FULVIO APARECIDO SANTOS ALVES, THAMIRES DE BRITO CAMARGO, GABRIELA AGUIAR VICENTE, DENNYS RIBEIRO DOS SANTOS, VICTOR PIANA DE ANDRADE, GENIVAL BARBOSA DE CARVALHO

**Affiliations:** 1 - AC Camargo Cancer Center - São Paulo - SP - Brasil

**Keywords:** Interdisciplinary Communication, Decision-making, Pancreatic Neoplasms, Comunicação Interdisciplinar, Tomada de Decisões, Neoplasias Pancreáticas

## Abstract

**Objective:**

the recommendations of the decisions made by the Tumor Board (TB) should be followed to identify barriers that may interfere with the execution of the previously decided, best care for the patient. The aim of this study is to assess whether the TB conduct decision was performed in patients with pancreatic tumors, their life status 90 days after the TB decision, and to analyze the reasons why the conduct was not performed.

**Methods:**

we conducted a retrospective study with patients with pancreas tumors, evaluated between 2017 and 2019. We collected data on epidemiological status, whether the TB procedure was performed, the reason for not performing it, life status 90 days after the TB decision, and how many times each patient was discussed at a meeting. We compared categorical variables using the chi square test, numerical variables were presented as means and standard deviation.

**Results:**

we studied 111 session cases, in 95 patients, 86 (90.5%) diagnosed with cancer. After 90 days of TB, 83 patients (87.37%) remained alive, 9 had (9.47%) died, and 3 (3.16%) were lost to follow-up. The TB decision was not observed in 12 (10.8%) cases and the reasons were: 25% (3) for loss of follow-up, 8.33% (1) for patient refusal, and 66.67% (8) due to clinical worsening. The cases of patients with metastases had a lower rate of TB conduct compliance (p=0.006).

**Conclusions:**

the TB conduct was performed in most cases and the most evident reason for non-compliance with the conducts is the patient’s clinical worsening.

## RESUMO

**Objetivo:**

as recomendações das decisões em Tumor Board (TB) deveriam ser acompanhadas para identificar barreiras que possam interferir na execução do melhor cuidado para o paciente decidido previamente. O objetivo do estudo é avaliar se a decisão de conduta em TB foi realizada em pacientes com tumores pancreáticos, o status de vida 90 dias após TB e analisar os motivos pelos quais a conduta não foi realizada.

**Métodos:**

estudo retrospectivo com pacientes com tumores de pâncreas, avaliados entre 2017 a 2019. Dados epidemiológicos, se a conduta de TB foi realizada, o motivo da não realização, o status de vida em 90 dias após decisão de TB e quantas vezes cada paciente foi discutido em reunião foram coletados. As variáveis categóricas foram comparadas pelo teste de qui-quadrado; variáveis numéricas foram apresentadas como médias e desvio padrão.

**Resultados:**

111 casos, 95 pacientes, 86 (90,5%) com diagnóstico de câncer. Após 90 dias de TB, 83 pacientes (87,37%) permaneceram vivos, 9 pacientes (9,47%) faleceram e 3 (3,16%) perderam o seguimento. A conduta do TB não foi realizada em 12 (10,8%) dos casos e os motivos foram: 25% (3) por perda de seguimento, 8,33% (1) por recusa do paciente e 66,67% (8) devido à piora clínica. Os casos de pacientes com metástases tiveram menor execução de conduta de TB (p=0,006).

**Conclusões:**

a conduta do TB é realizada na maior parte dos casos e o motivo mais evidente para o não cumprimento das condutas é a piora clínica do paciente.

## INTRODUCTION

The diversity of treatment options and diagnostic methods for cancer in recent years, due to the advancement of technology and the abundance of multimodal therapies, results in therapeutic plans that are often subspecialized^1^. Therefore, the interdisciplinary decision in the care of cancer patients is indispensable and influences clinical effectiveness^2^. Treatment strategies discussed in an interdisciplinary meeting, decided together with specialties, such as surgery, clinical oncology, radiotherapy, radiology, pathology, among other groups, result in the choice of targeted therapy for patients who do not fit the usual protocols due to the complexity of the disease, in addition to reducing variation in practice standards, helping in the judicious use of health resources^3^.

In this scenario of highly complex diseases, pancreatic cancer is evident, associated with an unfavorable prognosis, highlighted by the close parallel between the incidence of the disease and mortality^4^. Despite advances in the detection and treatment of pancreatic cancer, the 5-year survival rate is still only 9%, being one of the most lethal malignancies, requiring a model that helps in better care management, such as interdisciplinary meetings^5^. 

The interdisciplinary Tumor Board (TB) brings several benefits to better management of the care of patients who have difficult-to-manage diseases such as pancreatic cancer. This meeting model emerged with an educational focus in the 1980s, and evolved from greater hospital investments in the United States, which helped in the sharing of information between specialists, greater visibility of clinical research, and improved treatment strategies, ensuring better quality of patient care^6^. Currently, TB is used in many developed countries as an indicator of quality of service in cancer care^7^. Studies have shown that the care provided according to the patient’s clinical conditions, treated individually, and with the high quality of scientific guidelines, which conceptually occurs in the TB discussions, has resulted in better treatment, and may cause a decrease in the use of health resources^8^ ^,^ ^9^.

It is conceptualized as an interdisciplinary forum for the definition of oncological management in complex cases and without established protocols, representing one of the pillars of cancer centers in the world^10^, supporting the integration mechanism between Teaching, Research, and Assistance (proposed in the Cancer Center Program). An environment that generates hypotheses that can help in the evolution of cancer treatment, when combined with clinical and translational research, aims to establish the best treatment option (based on scientific evidence, knowledge and experience of specialists, national and international guidelines. and institutional protocols)^2^.

The benefits of TB meetings suggest an increase in the survival rate, educational opportunities, as it is associated with a more adequate staging classification, and, consequently, higher precision in the treatment plan^6^ ^-^ ^9^. 

Although clinical decisions made in TB patients are based on national and international scientific guidelines^9^, obstacles can influence the implementation of recommended conducts in TB patients. Therefore, these decisions must be followed up to identify which difficulties reflect the conduct not having been carried out. For example, a retrospective study, in Bristol, UK, evaluated whether 201 procedures decided in TB colorectal cancer were performed in the 157 analyzed patients, showing that only 10% of the decisions were not complied with, and the main reasons for non-execution were related to comorbidity, in 9 (40%) of the cases, and patient choice in 7 (35%)^11^.

Due to the complexity of pancreatic cancers, which demand broad interdisciplinary treatments, with constant reassessments of the initial plan, it is essential to assess the outcome of the cases discussed, for the creation of new protocols and evaluation of conducts. Currently, there are few studies on the outcome of conducts recommended by TB and there is no follow-up on whether the recommendation was carried out and or on its effectiveness for treatment. Knowing the reasons for non-compliance with the TB recommendations can help in interventions that improve the outcome of the conduct and prevent barriers that may interfere with the execution of the best care for the patient discussed in a meeting of specialists. Therefore, the aim of this study is to assess whether TB management decisions were followed through in patients with pancreatic neoplasms, analyzing the reasons why they have not been carried out, and the patients’ status 90 days after the TB session. 

## METHODS

We conducted a retrospective study, with patients diagnosed with malignant neoplasm of the pancreas and other pancreatic diseases, discussed at TB meetings from September 2017 to September 2019, through information collected from the electronic medical records. The study was carried out at a Cancer Center in the city of São Paulo, which has 14 TB clusters (breast tumors, skin tumors, hematological neoplasms, bone tumors and sarcomas, gynecological tumors, lung and chest tumors, upper digestive tract tumors, colorectal tumors, central nervous system tumors, head and neck tumors, urological tumors, pediatric tumors, pituitary and endocrinology tumors, and vascular and molecular tumors), organized in weekly forums lasting between one and two hours, with audiovisual resources.

We analyzed patients’ demographic and clinical data, whether the course of action decided on in the TB was performed, the reason for not performing it, the patient’s life status at 90 days after the interdisciplinary decision, date of death or last follow-up, and how many times each patient was discussed at a meeting. We defined the conduct as completely followed when the recommendation by the TB was fully carried out by the medical team; partially followed, when the conduct was not completely performed due to some obstacle; and not performed, when the no part of the recommended actions was followed by the interdisciplinary committee or there were no hospital records after the Tumor Board decision.

This study is part of the project entitled “Epidemiology and clinical outcomes of patients with tumors of the gastrointestinal tract discussed on a tumor board”, approved by the Ethics in Research Committee in September 2020, nº 2905/20.

The studied variables were the International Classification of Diseases (ICD), sex, diabetes mellitus (DM), hypertension (SAH), smoking, alcohol consumption, Eastern Cooperative Oncology Group (ECOG) Performance Status, Tumor Board doubt, metastases, therapeutic intent, paying source, requesting staff, age, and the ones associated with carrying out the conduct. We present categorical variables as absolute frequencies and simple ratios (percentages), we compared the variables’ distributions with the chi square test. We present numerical variables as mean and standard deviations, medians, and interquartile ranges, and compared them using the Mann Whitney U test.

## RESULTS

In the period from September 2017 to September 2019, 4,550 cases were discussed in the 14 TB centers of the institution. Of these, 886 (19.5%) were presented in the TB of Tumors of the Upper Digestive System, of which 111 cases (12.4%) were from patients with pancreatic diseases, corresponding to 95 patients (15 patients were discussed more than once in the analyzed period).

Of the 95 patients in the study, 50 (52.6%) were female, 86 (90.5%) were diagnosed with cancer, and 9 (9.5%) with other diseases of the pancreas, such as cysts and nodules. The age of the patients ranged from 17 to 88 years, with a mean of 62.5 years.

Among the comorbidities and habits evaluated, diabetes mellitus (DM) and systemic arterial hypertension (SAH) were present in 38 (40%) patients. Ten patients (10.5%) were smokers and three (3.2%) were alcoholics. As for performance status, 66 (59.5%) were classified as ECOG 0, 33 as ECOG 1 (29.7%), nine as ECOG 2 (8.1%), and three (2.7%) as ECOG 3. Of these patients, 78 (70.3%) had metastases (Table 1).

**Table 1:** Epidemiological profile of patients who were discussed in the TB.

Variables	n=95
Age, years - mean ± SD	62.5 ± 14.1
Sex	
Female	50 (52.6%)
Male	45 (47.4%)
Comorbidities	
SAH	38 (40%)
DM	38 (40%)
Habits	
Smoking - active	10 (10.5%)
Ex-smoker	24 (25.3%)
Alcoholism - active	3 (3.2%)
Ex-alcoholic	5 (5.3%)
Diagnosis (ICD)	
86 - other diseases of the pancreas	10 (9%)
C25 - malignant neoplasm of the pancreas	101 (91%)
Metastasis	
Yes	78 (70.3%)
No	33 (29.7%)
ECOG	
0	66 (59.5%)
1	33 (29.7%)
2	9 (8.1%)
3	3 (2.7%)

The number of times the patient was discussed in TB was also evaluated, in which 80 (84.2%) patients were discussed only once, 14 (14.7%) twice, and only one case was discussed three times.

Of the 111 cases discussed in the TB, 101 (91%) were brought up with doubts about therapeutic management and 10 (9%) due to doubts about diagnosis. The team that most requested cases to be discussed in a meeting was the oncology surgery team (n=92, 82.9%), followed by clinical oncology (n=17, 15.3%), and radiotherapy (n=2, 1.8%). When we analyzed the intention of the Tumor Board’s recommendation, most cases were of curative intent (n=63, 56.8%), followed by palliative one (n=48, 43.2%). The sources of payment were health insurance (n=83, 74.8%), public health system (n=23, 20.7%), and private (n=15, 13.5%). The TB conduct was performed completely in 98 (89.9%) of the cases, partially in one (0.90%) case, and in 12 (10.81%) cases the recommendations were not followed.

Of the 12 non-compliant cases, the reasons were: loss to follow-up (three), patient refusal (one), and clinical worsening (eight), as shown in Table 2.

**Table 2:** Characteristics of the cases discussed in the TB.

Variables	n=111
Case doubt	
Therapeutic Conduct	101 (91%)
Diagnosis	10 (9%)
Requesting team	
Oncological Surgery	92 (82.9%)
Clinical Oncology	17 (15.3%)
Radiotherapy	2 (1.8%)
Therapeutic Intent	
Curative	63 (56.8%)
Palliative	48 (43.2%)
Paying source	
Public Health System	83 (74.8%)
Health insurance	23 (20.7%)
Private	15 (13.5%)
Conduct performed?	
None	12 (10.8%)
Partially	1 (0.9%)
Completely	98 (88.3%)
Reasons for not performing the conduct	
Follow-up loss	3 (7.7%)
Clinical worsening	8 (7.2%)
Patient refusal	1 (0.9%)

The variables ICD, sex, DM, SAH, smoking, alcohol consumption, ECOG, Tumor Board doubt, therapeutic intention, paying source, requesting team, and age displayed no statistically significant association.

**Graph 1:**
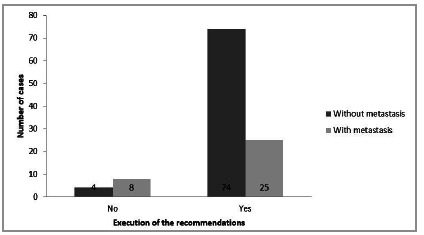
Correlation between patients with metastasis and execution of the recommendations.

When comparing the execution of the TB procedure and the presence of metastasis by the chi-square test, we observed that cases with metastasis had showed less compliance with the recommendations (p=0.006) (Graph 1).

After 90 days of the TB meeting, 83 (87.37%) patients remained alive, nine (9.47%) had died, and three (3.16%) were lost to follow-up.

## DISCUSSION

In Brazil, pancreatic cancer is responsible for approximately 2% of all types of cancer and 4% of all deaths caused by the disease^12^. Due to the high complexity, the advancement of the disease and the restriction of treatment options, a the joint conduct decision becomes necessary on several occasions, as the ones performed in the TB forums, which allow the exchange of specialists’ experiences, based on international guidelines that promote better care management^13^. In this study, we analyzed 86 patients (90.5%) diagnosed with pancreatic cancer and 9 (9.5%) with other diseases of the pancreas, such as cysts and nodules. 

The Tumor Board recommendations help in the approach of difficult-to-manage diseases with the support of several specialists, therefore promoting better best patient management. To ensure this care, some studies evaluated adherence to the conduct, such as a study carried out in Saudi Arabia in 2016. Conducted prospectively, this cohort in King Abdulaziz Medical City evaluated the consistency of the recommendations of Gastrointestinal TB with international guidelines from the National comprehensive Cancer Network (NCCN), and adherence of physicians involved in patient care to TB recommendations, as well as the impact on patient management. Of the 104 patients included, 24 recommendations (23%) were made. Adherence to National guidelines comprehensive Cancer Network was observed in 97% of the total recommendations. During a period of three months after the presentation of the TB case, most of the recommendations (87%) were carried out. The authors concluded that the existence of TB improves adherence to recommended guidelines and has an impact on patient care management in approximately one third of patients^14^.

Other authors substantiated the significant increase in the survival rate of cancer patients, regardless of tumor site, discussed in an interdisciplinary TB meeting^15^.

According to a randomized controlled clinical trial, a survival rate of more than two years was found in patients with Lung Cancer who were followed up by an interdisciplinary team, in relation to patients with the same diagnosis not submitted to the same approach. This corroborates the effectiveness and benefits of interdisciplinary discussions on TB, one of the pillars of a Cancer Center^16^.

Interdisciplinary forums for discussion of conduct are means that allow the patient to be offered a better decision-making process, from the perspective of several specialists. The act of all professionals being together and being enable opinions to converge aimed at the diagnosis or assertive treatment, even for patients with metastases, contributes to better control of signs and symptoms, suggesting an increase in quality of life and satisfaction with the proposed treatment. For the medical professional, it is also a forum that provides security, as it shares the responsibility and seriousness of discussing and proposing treatment for a complex case that does not fit current protocols. The constancy of these sessions contributes to the continuing medical education of the team, as well as offering the opportunity to improve protocols and institutional processes.

## CONCLUSION

The TB recommendations were carried out in most cases and the most obvious reason for non-compliance with the conducts is the clinical worsening of the patient. Cases with metastases are susceptible to clinical worsening, thus implying non-compliance with the proposed treatment sequence.
